# Supplementary material for: Evolution of China’s interaction with Middle Eastern countries under the Belt and Road Initiative
Source: PLoS One. 2023 Nov 9;18(11):e0293426. doi: 10.1371/journal.pone.0293426 (PMC10635491; doi:10.1371/journal.pone.0293426)
Supplement: Attachment — Submitted filename: Response to peer review.docx [file pone.0293426.s001.docx]

Thanks to the corresponding author and research team. It is clear that a lot of effort has been put into writing the manuscript.

1. Based on Formula “**Interactive Relationship Types under Reciprocity**”, the relationship between Iran and China; what is the position of 𝑅𝑗? be explained?

𝑅𝑗=1

𝑅𝑗 > 0.01

𝑅𝑗 < -0.01

2. Page 11; line 230 refers to Iran-China relations in political, diplomacy, economy, culture, and other fields, which has led to multi-level cooperation? The question that arises is that; Considering the many cultural and religious differences between the two countries, how were they able to overcome the differences and form multi-level cooperation?
3. Considering that China adheres to non-interference policy in the internal affairs of other countries; with what policy did he help to improve relations between Iran and Saudi Arabia? So that he can finally transform relations from the "single cooperation" approach to the "double cooperation" model?
