# Supplementary material for: Evolution of China’s interaction with Middle Eastern countries under the Belt and Road Initiative
Source: PLoS One. 2023 Nov 9;18(11):e0293426. doi: 10.1371/journal.pone.0293426 (PMC10635491; doi:10.1371/journal.pone.0293426)
Supplement: Attachment — Submitted filename: Response to Reviewers.docx [file pone.0293426.s002.docx]

Subject: Response to Reviewer Comments – Manuscript ID: PONE-D-23-26208

Dear Reviewer 1 and Reviewer 2,

I hope this email finds you well. I want to express my sincere appreciation for your time and effort in reviewing my manuscript titled Evolution of China's Interaction with Middle Eastern Countries under the Belt and Road Initiative (Manuscript ID: PONE-D-23-26208).

Reviewer 1:

1. In the introduction section, in addition to the importance of the Middle East, it is better to refer to relations China's international and strategic relationship with other regions of the world in the past and present is also mentioned, this will engage the reader's mind more to understand your study.

We sincerely thank the reviewer for careful reading. As suggested by the reviewer, we have incorporated a discussion on China's international strategies and developmental processes across various regions worldwide in the opening of this article.

2. Although the Goldstine index is briefly mentioned in the methodology section, It is better to refer to the use of this index in previous studies. providing clear examples of the indexs application would be helpful. (more emphasis on explaining its parameters will enhance the validity and reliability of the analysis )

This is a valuable suggestion, and we have supplemented information on the use cases of Goldstein scores in the article, specifically at line 143. Additionally, we have included references 36 and 37 to support this update.

[36]	Li, YH; Jian, Z; Zhao, LX. How political conflicts distort bilateral trade: Firm-level evidence from China. Journal of Economic Behavior & Organization. 2021;183, 233-249

[37]	Zhang, X., Zhang, X., Zhang, L., et al. Spatial-temporal Evolution Characteristics of China's Geopolitical Relations with Belt and Road Countries. World Geography Research, 2023;32(09), 17-27.

3. To enhance the clarity and conciseness of the discussion section, I suggest the author critically each reference. in this case, adding references based on similar studies can be valuable.

Thank you for your valuable suggestions. Given the multitude of countries involved in this study and the limited existing research, I have made concerted efforts to comprehensively reorganize the discussion section. Additionally, I have included references 41, 42, and 43 to further support and enrich the context. Your insights have been instrumental in refining the content of the paper.

[41]	YU Zhen. Third-Party Market Cooperation between China and Middle Eastern Countries under the Background of the Belt and Road Initiative. International Relations Studies, 2023;2023(04), 59-81+157-158.

[42]	Chaziza, M. China's Economic Diplomacy Approach in the Middle East Conflicts. China Report. 2019;55 (1), pp.24-39

[43]	Almujeem, NS. GCC countries' geoeconomic significance to China's geopolitical ends. Review Of Economics And Political Science. 2021;6(4), 348-363

Reviewer 2:

1. Based on Formula “Interactive Relationship Types under Reciprocity”, the relationship between Iran and China; what is the position of 𝑅_𝑗_? be explained?

We apologize for not clearly stating this concept in the article. Let 𝑅_𝑗_ represent the type of interaction between China and Iran. When 𝑅_𝑗_ > 0.01, it indicates that the impact of cooperative events between China and Iran, as a proportion of the total impact of China's cooperation events in the Middle East, significantly exceeds the proportion of conflict events' impact. In this scenario, the interaction between China and Iran is classified as cooperation-dominant. Conversely, when 𝑅_𝑗_ < -0.01, China's interaction with Iran is conflict-dominant, meaning the impact of conflict events surpasses that of cooperative events. For cases where -0.01 ≤ 𝑅_𝑗_ ≤ 0.01, the interaction between China and Iran is considered balanced.

Over the decade from 2013 to 2022, more than half of the Middle Eastern countries demonstrated a cooperative-dominant interaction with China. As depicted in Figure 5, China and Iran exhibited a cooperation-dominant relationship for six out of the ten years, with 𝑅_𝑗_ greater than 0.01, and conflict-dominant for four years with 𝑅_𝑗_ less than -0.01. Consequently, over the past decade, the interaction between Iran and China, in comparison to the overall interactions in the Middle East, tends to be characterized as cooperation-dominant in this study.

2. Page 11; line 230 refers to Iran-China relations in political, diplomacy, economy, culture, and other fields, which has led to multi-level cooperation? The question that arises is that; Considering the many cultural and religious differences between the two countries, how were they able to overcome the differences and form multi-level cooperation?

Thank you for your thoughtful question. We sincerely apologize if any part of the statement lacked clarity. The enduring history of amicable interactions between Iran and China, despite cultural and religious distinctions, has been characterized by continuous economic collaboration. In recent years, propelled by international dynamics, the political and diplomatic ties between our two nations have notably strengthened, forming a robust alliance across various fronts. To eliminate any potential misunderstandings, I have carefully revised ambiguous expressions in the original text. Your inquiry is greatly appreciated.

3. Considering that China adheres to non-interference policy in the internal affairs of other countries; with what policy did he help to improve relations between Iran and Saudi Arabia? So that he can finally transform relations from the "single cooperation" approach to the "double cooperation" model?

We appreciate your attention to the matter. Regarding China's influence on the improved relations between Iran and Saudi Arabia, it's crucial to note that the primary driver for reconciliation between these two nations arises from their intrinsic willingness to mend ties.

Examining the respective situations in Iran and Saudi Arabia, the Middle East dynamics, and the global landscape, the years spanning from 2016 to 2020 were not conducive to a resolution due to divergent positions in conflicts such as Syria and Yemen, as well as varied approaches to addressing the "Islamic State" threat. However, starting in 2020, both parties embarked on a gradual path towards easing tensions, culminating in a nearing reconciliation in 2023.

China, upholding a policy of non-interference in the internal affairs of sovereign states, emerged as an external mediator acceptable to both sides. This diplomatic role has effectively facilitated the improvement of bilateral relations. Furthermore, as a major global consumer market and a proponent of the Belt and Road Initiative, China has engaged in cooperative efforts with Iran and Saudi Arabia.

I have carefully considered each of your comments and suggestions during the revision process. The manuscript has been updated accordingly to address the concerns raised and to improve its overall quality.

I appreciate the insightful feedback provided by both of you, which has undoubtedly enhanced the clarity and robustness of the paper. I believe these revisions align well with the standards of PLOS ONE.

I look forward to any additional feedback you may have and would be grateful for your further guidance to ensure the manuscript meets the journal's expectations.

Thank you once again for your valuable contributions to the improvement of my work. Your dedication to maintaining the high standards of PLOS ONE is truly commendable.

Best regards,

Yang Xiaolu

yxl5011@email.swu.edu.cn
